# Mortality and health-related quality of life in prevalent dialysis patients: Comparison between 12-items and 36-items short-form health survey

**DOI:** 10.1186/1477-7525-10-46

**Published:** 2012-05-06

**Authors:** Tone Brit Hortemo Østhus, Valjbona Tiric Preljevic, Leiv Sandvik, Torbjørn Leivestad, Inger Hilde Nordhus, Toril Dammen, Ingrid Os

**Affiliations:** 1 Department of Nephrology, Oslo University Hospital Ullevål, Kirkeveien 166, Oslo 0407, Norway; 2 Faculty of Medicine, University of Oslo, Oslo, Norway; 3 Department of Psychiatry, Oslo, Norway; 4 Section of Epidemiology and Statistics, Oslo University Hospital Ullevål, Oslo, Norway; 5 Institute of Immunology, Oslo University Hospital Rikshospitalet, Oslo, Norway; 6 Norwegian Competence Center for Sleep Disorders, Haukeland University Hospital, Bergen, Norway; 7 Department of Clinical Psychology, Faculty of Psychology, University of Bergen, Bergen, Norway

**Keywords:** Chronic kidney disease, Dialysis, Health-related quality of life, Mortality, Physical component summary score, SF-12 and SF-36

## Abstract

**Background:**

To assess health- related quality of life (HRQOL) with SF-12 and SF-36 and compare their abilities to predict mortality in chronic dialysis patients, after adjusting for traditional risk factors.

**Methods:**

The Short-Form Health Survey (SF-36) with the embedded SF-12 was applied in 301 dialysis patients cross-sectionally. Physical and mental component summary (PCS-36, MCS-36, PCS-12, and MCS-12) scores were calculated. Clinical and demographic data were collected. Mortality (followed for up to 4.5 years) was analyzed with Kaplan Meier plots and Cox proportional hazards, after censoring for renal transplantation. Exclusion factors were observation time <2 months (n = 21) and missing component summary scores (n = 10 for SF-36; n = 28 for SF-12), thus 252 patient were included in the analyses.

**Results:**

In 252 patients (60.2 ± 15.5 years, 65.9% males, dialysis vintage 9.0, IQR 5.0-23.0 months), mortality during follow-up was 33.7%.(85 deaths). Significant correlations were observed between PCS-36 and PCS-12 (*ρ* = 0.93, *p* < 0.001) and between MCS-36 and MCS-12 (*ρ* = 0.95, *p* < 0.001). Mortality rate was highest in patients in the lowest quartile of PCS-12 (*χ*^2^ = 15.3, *p* = 0.002) and PCS-36 (*χ*^2^ = 16.7, *p* = 0.001). MCS was not associated with mortality. Adjusted hazard ratios for mortality were 2.5 (95% CI 1.0-6.3, PCS-12) and 2.7 (1.1 – 6.4, PCS-36) for the lowest compared with the highest (“best perceived”) quartile of PCS.

**Conclusion:**

Compromised HRQOL is an independent predictor of poor outcome in dialysis patients. The SF-12 provided similar predictions of mortality as SF-36, and may serve as an applicable clinical tool because it requires less time to complete.

## Introduction

Despite advances in dialysis treatment and improvements in the management of traditional cardiovascular risk factors, mortality rates for patients with end-stage renal disease (ESRD) on chronic dialysis remain unacceptably high. For patients with ESRD in Europe and the United States, survival rates after initiation of dialysis treatment are 81.1% and 80.4%, respectively, at one year and 38.2% and 35.8%, respectively, after five years [1,2]. The established predictors of mortality in patients on dialysis include low serum albumin [3], hemoglobin [4], and increasing age [5]. In addition, patients rejected for renal transplantation are at special risk for lethal outcome [6]. Studies have suggested that high mortality rates might be reduced by improving the quality of dialysis, control of phosphates, normalization of serum albumin, and correction of renal anemia [7-9]. However, despite data that indicates that these quality measures in dialysis are improving, mortality rates have not improved in parallel [10].

Recent studies have suggested that a poor health-related quality of life (HRQOL) was strongly related to increased risk of mortality in patients on dialysis [11-17]. Thus, although HRQOL is typically used to gain information about patient well-being, it may also indicate the risk of important outcomes, like death.

The medical outcome survey Short Form 36 (SF-36) has been widely used and validated as an HRQOL assessment tool in general populations and in patients with ESRD [11,12,18,19]. SF-12, a shortened version of the SF-36 questionnaire has recently been introduced, but it has been rarely used for patients on dialysis, despite the advantage that it comprises only one third of the items compared to SF-36 [20]. The SF-12 was recently employed in a U.S. study on a large cohort of 44 395 patients on dialysis. Those authors concluded that the physical (PCS) and mental composite summary (MCS) scores based on the SF-12 were valid in this patient group. Furthermore, they showed that the prognostic information with regard to mortality was similar to that of the SF-36 [21]. To the best of our knowledge, the SF-12 has not been specifically validated in Europe for patients on dialysis; nor has any European study examined whether the SF-12-based HRQOL scores might be predictive of mortality. As the self-perceived HRQOL has been shown to diverge between countries, it is important to undertake studies of HRQOL in different countries. We suggest that the component summary scores from SF-12 and SF-36 are highly correlated. Furthermore, we hypothesized that self-assessed HRQOL based on the SF-12 and the SF-36 would provide similar predictions of mortality in patients on dialysis.

The objectives of the present study were to assess HRQOL with SF-12 and SF-36 and compare their abilities to predict mortality in chronic dialysis patients, after adjusting for traditional risk factors.

## Methods

### Study patients and design

**Figure 1:**
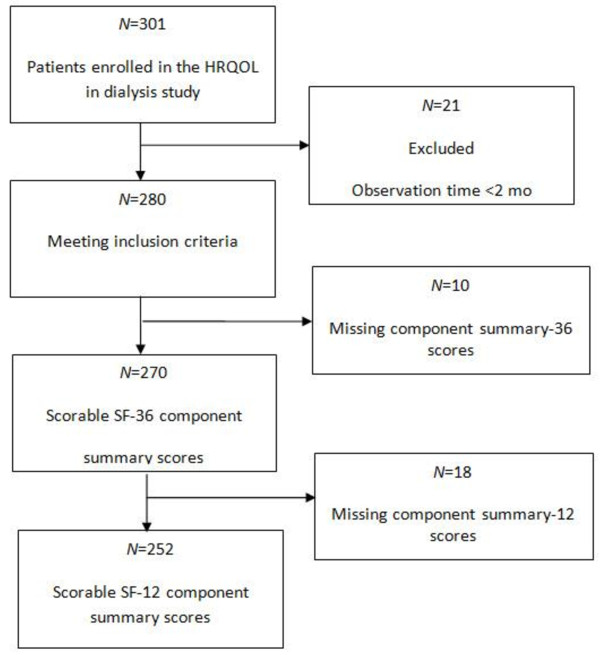
Participant flow-chart.

In this observational prospective cohort study, the primary aim was to determine the association between HRQOL and mortality. We included a total of 301 prevalent dialysis patients (243 on hemodialysis and 58 on peritoneal dialysis) from ten dialysis clinics in Norway. Baseline HRQOL data were previously reported [22]. All adult patients (≥18 years old) that had received hemodialysis (HD) or peritoneal dialysis (PD) for more than 2 months were screened for study participation. Patients were excluded from the study when they were hospitalized during the investigation period; however, they could be enrolled four weeks or more after hospital discharge, if they were in stable clinical condition. Patients were excluded that displayed severely impaired cognitive function, psychosis, or drug abuse. The study required adequate Norwegian language skills. Signed informed consent was required for enrollment, after patients received oral and written information about the study. Detailed information regarding mortality and cause of death was obtained from the Norwegian Renal Registry. Patients were enrolled in the study from August 2005 to February 2007, and they were followed until January 2010. The recruitment process was described in detail previously [22]. Briefly, of the 416 patients considered eligible for the study, 326 patients consented to study participation, and 301 could be enrolled (enrollment rate of 72.4%). Patients with observation time less than 2 months were excluded from the survival analyses (Figure 1), and the time of renal transplantation was censored. To ensure standardized conditions, self-administered questionnaires were completed during the regular hemodialysis sessions for patients on HD or during the scheduled visit at the outpatient clinic for patients on PD. Study nurses and physicians were specifically trained in applying the study instruments.

The National and Regional Committees for Research Ethics in Norway approved the study protocol, and permission was obtained from the National Data Inspectorate.

### Demographic and clinical data at baseline

Demographic data including age, gender, and work status were collected from reviews of hospital charts and/or by directly questioning the patients. The cause of renal failure, dialysis modality, dialysis vintage, comorbidities, and laboratory data were gathered from medical records. Comorbidity was measured with the modified Charlson comorbidity index (CCI) [23]. The CCI is a composite score of 17 multiple comorbid conditions (e.g., coronary artery disease and congestive heart failure) and age. In this study, CCI was calculated without age, because we intended to evaluate the effect of age as a separate factor in the multivariate analysis.

### Assessment of HRQOL

The Medical Outcome Study 36-item Short-Form health survey (SF-36) [18] was applied to assess the general dimensions of HRQOL. A validated Norwegian version of the SF-36 version 1 was applied [24]. The physical component summary (PCS-36) and the mental component summary (MCS-36) scores were derived from eight SF-36 subscales, as described by Ware et al. [25]. These scores ranged from 0 to 100, where a higher score represented better self-assessed health. The embedded SF-12 comprises 12 questions from the SF-36, and the component summary scores of SF-12 were calculated with the algorithm from the KDQoL working group (http://gim.med.ucla.edu/kdqol/downloads). The PCS-36 and PCS-12 included physical functioning, physical role limitation, and bodily pain; the MCS-36 and MCS-12 included mental health, social functioning, and emotional role limitation. General health and vitality were incorporated in all component summary scores. Recent reports showed strong correlations between the PCS-36 and PCS-12 and between MCS-12 and MCS-36 in patients with ESRD [21].

### Statistical analyses

Clinical, demographic, and HRQOL variables were expressed as means and standard deviations (SDs), or medians with interquartile ranges (IQR), when data were skewed. Categorical variables were measured as frequencies and percentages. The one-way analysis of variance (ANOVA) or Kruskall-Wallis tests for skewed data were used to compare continuous variables between more than two groups; the Student’s *t*-test or the Mann–Whitney test for skewed data was applied for comparisons between two groups. The chi-square test was used to compare categorical variables. HRQOL component summary scores (PCS-36, MCS-36, PCS-12 and MCS-12) were divided into quartiles, with equal number of patients in each quartile group (n = 63). Although HRQOL is considered a continuous variable, we implemented quartiles to reveal clinically significant differences. Kaplan-Meier curves were applied to compare survival rates between groups with different HRQOL quartile scores. Cox proportional hazard models were used to estimate the unadjusted and adjusted hazard ratios (HRs) of death for groups with different HRQOL quartile scores, and for changes in continuous HRQOL scales by one-unit increments. HRs are presented with 95% confidence intervals. In quartile analyses, the upper quartile (best perceived state) was used as the reference level. All demographic and clinical variables listed in Table 1 were set as independent variables in separate univariate Cox regression analyses to identify variables significantly associated with death; variables with p <0.2 were entered into the adjusted Cox regression model as covariates. Spearman’s correlations were performed to determine associations between the demographic and clinical variables and HRQOL component summary scores (PCS-36 and MCS-36). When a variable was significantly associated (p < 0.2) with both death and the PCS-36 or MCS-36 score, it was considered a potential confounder. When the Spearman’s correlation coefficient between two potential confounders was outside the interval −0.70, 0.70, one was excluded.

**Table 1:** Demographic and clinical baseline data for the study patients (n = 252), according to physical and mental component summary-36 score quartiles

	**All patients**	**Physical component summary-36 score quartiles**					**Mental component summary-36 score quartiles**				
		**Q**_**1**_**Range: 9.6-30.0**	**Q**_**2**_**Range: 30.1-35.6**	**Q**_**3**_**Range: 35.7-44.4**	**Q**_**4**_**Range: 44.5–58.2**	**P -value**	**Q**_**1**_**Range: 16.9-39.2**	**Q**_**2**_**Range: 39.3-49.0**	**Q**_**3**_**Range: 49.1-55.6**	**Q**_**4**_**Range: 55.7-70.7**	**P - value**
N	252	63	63	63	63		63	63	63	63	
Age, yrs, (252)	60.2±15.5	60.8±12.4	64.8±14.9	58.1±17.3	57.2±16.0	0.027^p^	56.5±15.7	63.7±15.3	60.0±17.0	60.6±13.0	0.071^p^
Male gender,%, (n=252)	65.9 (166)	58.7 (37)	68.3 (43)	69.8 (44)	66.7 (42)	0.563^2^	61.9 (39)	68.3 (43)	77.8 (49)	55.6 (35)	0.056^2^
Current smoker, %, (n=252)	25.8 (65)	33.3 (21)	23.8 (15)	22.2 (14)	23.8 (15)	0.466 ^2^	39.7 (25)	22.2 (14)	19.0 (12)	22.2 (14)	0.034^2^
**Work status, %, (n)**											
Able to work, (n=235)	12.3 (29)	8.5 (5)	5.2 (3)	12.1 (7)	23.3 ( 14)	0.016 ^2^	6.7 (4)	15.3 (9)	12.7 (7)	14.8 (9)	0.460^2^
Disable to work, (n=235)	51.5 (121)	64.4 (38)	43.1 (25)	50.0 (29)	48.3 (29)	0.118 ^2^	65.0 (39)	35.6 (21)	47.3 (26)	57.4 (35)	0.009^2^
Retired, (n=235)	36.2 (85)	27.1 (16)	51.7 (30)	37.9 (22)	28.3 (17)	0.02 ^2^	28.3 (17)	49.2 (29)	40.0 (22)	27.9 (17)	0.045^2^
**Cause of renal failure, %, (n)**											
Glomerulonephritis, (n=249)	20.5 (51)	14.3 (9)	20.6 (13)	19.4 (12)	27.9 (17)	0.311 ^2^	20.6 (13)	12.7 (8)	26.7 (16)	22.2 (14)	0.276^2^
Diabetic nephropathy, (n=249)	14.1 (35)	22.2 (14)	6.3 (4)	16.1 (10)	11.5 (7)	0.068 ^2^	15.9 (10)	14.3 (9)	16.7 (10)	9.5 (6)	0.663^2^
Hypertensive kidney disease, (n=249)	24.9 (62)	25.4 (16)	25.4 (16)	27.4 (17)	21.3 (13)	0.886 ^2^	25.4 (16)	25.4 (16)	23.3 (14)	25.4 (16)	0.991^2^
Other, (n=249)	40.6 (101)	38.1 (24)	47.6 (30)	37.1 (23)	39.3 (24)	0.613 ^2^	38.1 (24)	47.6 (30)	33.3 (20)	42.9 (27)	0.408^2^
**Clinical variables**											
Dialysis vintage, mo, (n=251)	9.0 (5.0, 23.0)	18.0 (6.0, 34.0)	9.0 (4.0, 20.0)	9.0 (5.0, 20.0)	7.0 (3.4, 16.8)	0.004^np^	10.0 (4.0, 23.0)	11.0 (5.0, 32.0)	10.0 (5.0, 17.3)	7.0 (4.0, 24.0)	0.352^np^
Previous graft failure, (251)	18.7 (47)	24.2 (15)	11.1 (7)	19.0 (12)	20.6 (13)	0.287 ^2^	22.2 (14)	17.5 (11)	21.0 (13)	14.3 (9)	0.661^2^
Accepted for renal transplantation, (n=252)	38.1 (96)	28.6 (18)	27.0 (17)	49.2 (31)	47.6 (30)	0.01^2^	36.5 (23)	33.3 (21)	42.9 (27)	39.7 (25)	0.718^2^
Peritoneal dialysis, (n=252)	20.2 (51)	23.8 (15)	14.3 (9)	27.0 (17)	15.9 (10)	0.221 ^2^	14.3 (9)	19.0 (12)	31.7 (20)	15.9 (10)	0.062^2^
Body mass index, kg/m^2^, (n=235)	24.9±4.9	23.6±5.0	25.5±4.8	24.9±4.6	25.5±5.0	0.135^p^	23.7±4.5	25.2±4.4	25.0±4.6	25.9±5.8	0.124^p^
Serum albumin, g/l, (n=246)	38.0±4.8	36.6±5.9	37.7±4.4	38.4±4.3	39.1±4.0	0.022^p^	38.7±3.9	37.8±4.4	38.1±5.5	37.4±5.2	0.503^p^
Hemoglobin, g/dl, (n=246)	12.1±1.5	12.0±1.4	12.1±1.5	12.2±1.5	12.2±1.4	0.87^p^	11.8±1.6	12.1±1.3	12.4±1.5	12.2±1.3	0.139^p^
Total cholesterol, mmol/L, (n=230)	4.2±1.1	4.0±1.2	4.1±1.1	4.5±1.1	4.2±1.1	0.103^p^	4.3±1.2	4.0±1.0	4.2±1.2	4.3±1.1	0.279^p^
**Comorbidity**											
Diabetes, %, (n=250)	26.4 (66)	31.7 (20)	28.6 (18)	24.2 (15)	21.0 (13)	0.537 ^2^	30.2 (19)	33.3 (21)	27.9 (17)	14.3 (9)	0.077^2^
CCI without age, (n=248)	4 (2, 5)	5 (4, 6)	4 (2, 4)	3 (2, 5)	3 (2, 4)	<0.001^np^	4 (3, 5)	4 (3, 5)	4 (2, 5)	3 (2, 5)	0.436^np^

*CCI* Charlson modified comorbidity index. Continuous variables are given as mean ± SD, when normally distributed, or as median (IQR), when skewed. P-values between the four groups are calculated based on parametric (ANOVA)^p^ , nonparametric (Kruskall Wallis)^np^, or Chi-squared^2^ statistics. Note: Numbers of complete data are given in parentheses for each variable.

To identify the most important covariates, all selected variables were entered into multivariate linear regression models with PCS-36 and MCS-36 as dependent variables. By backward variable selection, only variables with p <0.1 were analyzed further.

Age, dialysis vintage, and the Charlson comorbidity index were included in the final model as covariates. Due to the selection criteria, serum albumin was included in the model that examined the relationship between death and the PCS-36 or PCS-12 quartile score. Hemoglobin was included in the model that examined the relationship between death and the MCS-36 or MCS-12 quartile score. Gender was included as a covariate in the final model, despite the lack of significant associations with death. When a variable markedly deviated from a normal distribution, data were log-transformed (e.g., dialysis vintage) before inclusion into the regression model as a covariate [26].

The significance level was set to 5%. The data were analyzed with SPSS for Windows, version 16 (SPSS, Chicago, IL, USA).

## Results

Of the 301 patients enrolled in the study, 21 patients were excluded from the survival analysis due to short observation time (< 2 months). Ten patient SF-36 component summary scores were missing, and additionally 18 patient SF-12 component summary scores. Thus, data from 252 patients was analyzed (Figure 1). The follow-up time ranged from 2.8 to 4.5 years, with a median of 3.6 years (IQR 3.2 to 3.9). The time from study inclusion to death or kidney transplantation ranged from 0.2 to 4.3 years, with a median time of 1.5 years (IQR 0.9 to2.7). At the end of follow-up, 85 (33.7%) patients had died, and 122 (48.4%) patients had received a renal transplant.

**Figure 2:**
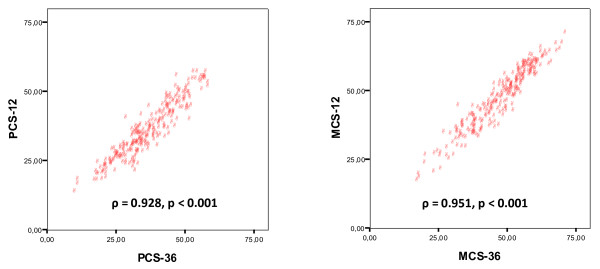
**Scatter plots display correlations between physical and mental component summary scores (PCS and MCS, respectively).** The scores were calculated from the SF-12 (ordinate) and SF-36 (abscissa) assessments of health related quality of life in patients on chronic dialysis (n = 252).

Highly significant correlations were observed between the PCS-36 and PCS-12 (r = 0.932, *ρ* = 0.928, p <0.001 for both, n = 252, Figure 2), and between the MCS-36 and MCS-12 (r = 0.953, *ρ* = 0.951, p <0.001 for both, n = 252, Figure 2).

Characteristics of the patients, grouped by quartiles of PCS-36 and MCS-36, are presented in Table 1. For the whole study population (n = 252), the mean scores for PCS-36 was 36.6 ±10.4 (range 9.6 - 58.2), the MCS-36 was 47.3 ±11.0 (16.9 -70.7), PCS-12 was 35.5 ± 9.9 (13.3 – 56.6), and MCS-12 was 46.9 ± 10.9 (16.7 -70.4). Age, dialysis vintage, serum albumin, and comorbidity differed between PCS-36 quartiles; age, smoking, and workability differed between MCS-36 quartiles (Table 1).

The most frequent causes of death were cardiovascular disease 42.4% (n = 36), sepsis 31.8% (n = 27), and malignant disease 14.1% (n = 12). Withdrawal from dialysis occurred in 4.7% (n = 4). In univariate Cox regression analyses (Table 2), mortality was significantly associated with age, current smoking, log transformed dialysis vintage, being rejected for renal transplantation, presence of diabetes and comorbidity score. In contrast, mortality was not associated with gender, dialysis modality, hemoglobin, previous graft failure, serum albumin, body mass index, or cholesterol.

**Table 2:** Impact of demographic and clinical variables on mortality in chronic dialysis patients (n = 252) during follow-up (median follow-up time 3.6 years), univariate associations are shown

	**Hazard ratio**	**95% CI**	**p-value**
Age, per year increment	1.026	1.009 – 1.044	0.002
Gender, male vs female	1.209	0.768 – 1.901	0.412
Currents smoking, yes vs no	1.772	1.125 – 2.790	0.014
**Work status**			
Able to work, yes vs no	0.510	0.186 – 1.398	0.191
Disable to work, yes vs no	1.030	0.659 – 1.610	0.896
Retired, yes vs no	1.162	0.743 – 1.819	0.510
**Cause of renal failure**			
Glomerulonephritis, yes vs no	1.015	0.571 – 1.804	0.959
Diabetic nephropathy, yes vs no	1.704	0.987 – 2.942	0.056
Hypertensive kidney disease, yes vs no	1.147	0.723 – 1.821	0.560
**Clinical variables**			
Dialysis vintage, per month increment	1.009	0.998 – 1-020	0.095
Log-dialysis* vintage, per unit increment	1.284	1.041 – 1.585	0.020
Previous graft failure, yes vs no	1.748	0.926 – 3.299	0.085
Rejected for renal transplantation, yes vs no	1.965	1.063 – 3.635	0.031
Dialysis modality, hemodialysis vs. peritoneal dialysis	1.091	0.632 – 1.883	0.755
Body mass index, per unit (kg/m^2^) increment	0.985	0.939 – 1.034	0.536
Albumin, per unit (g/l) increment	0.978	0.937 – 1.012	0.176
Hemoglobin, per unit (g/dl) increment	0.879	0.758 – 1.019	0.088
Cholesterol, per unit (mmol/l) increment	0.937	0.747 – 1.176	0.574
Diabetes, yes vs no	1.579	1.002 – 2.487	0.049
Charlsons modified comorbidity index without age, per unit increment	1.260	1.136 – 1.398	<0.001

*Log-transformed dialysis vintage.

Abbreviations: *CI* confidence interval.

**Figure 3:**
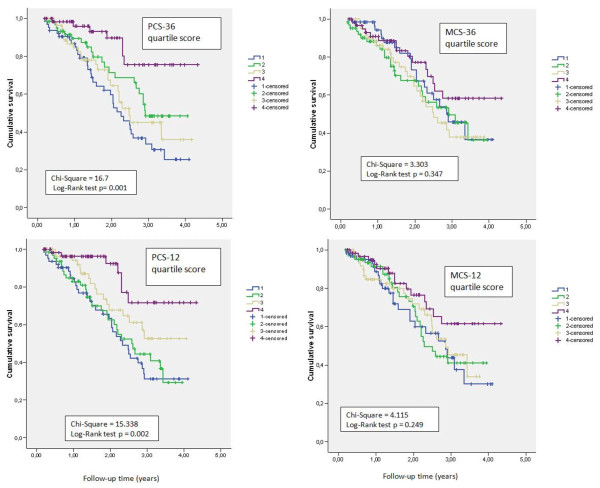
**Kaplan-Meier plots of mortality rates in quartiles (Q1-Q4) of physical (left, PCS) and mental (right, MCS) component summary scores.** Scores were calculated with the SF-36 (n = 252, upper panels) or SF-12 (n = 252, lower panels) assessments in patients on chronic dialysis.

Mortality rates were significantly different in the highest and lowest PCS-12 quartiles, based on the Kaplan Meier curves (Figure 3). A similar difference was observed for PCS-36 quartiles (Figure 3). In contrast, mortality rates were not different between quartiles for either the MCS-36 or MCS-12 (Figure 3, Table 3).

**Table 3:** Unadjusted and multi-adjusted hazard ratios (HRs) for mortality were assessed for patients on dialysis, grouped by physical and mental component summary (PCS-36, MCS-36, PCS-12, and MCS-12) quartile scores

	**PCS-36 quartile score**				**MCS-36 quartile score**			
	**Unadjusted HR (95% CI)**	**p-value**	**Adjusted HR (95% CI)**	**p-value**	**Unadjusted HR (95% CI)**	**p-value**	**Adjusted**^**B**^**HR(95% CI)**	**p-value**
Q^4^	1 (reference)		1 (reference)		1 (reference)		1 (reference)	
Q^3^	3.516 (1.508, 8.196)	0.004	2.495 (1.041, 5.976)	0.040	1.720 (0.911, 3.249)	0.095	1.262 (0.616, 2.584)	0.525
Q^2^	2.599 (1.104, 6.119)	0.029	1.741 (0.721, 4.205)	0.218	1.634 (0.857, 3.115)	0.136	1.460 (0.735, 2.898)	0.280
Q^1^	4.547 (2.016, 10.259)	<0.001	2.675 (1.126, 6.355)	0.026	1.365 (0.698, 2.667)	0.363	1.676 (0.845, 3.327)	0.714
	PCS-12 quartile score				MCS-12 quartile score			
Q^4^	1 (reference)		1 (reference)		1 (reference)		1 (reference)	
Q^3^	2.248 (0.932, 5.423)	0.072	1.658 (0.630, 4.365)	0.306	1.262 (0.616, 2.584)	0.525	1.671 (0.859, 3.250)	0.130
Q^2^	3.618 (1.584, 8.263)	0.002	2.423 (0.964, 6.087)	0.060	1.460 (0.735, 2.898)	0.280	1.746 (0.907, 3.61)	0.095
Q^1^	4.056 (1.789, 9.194)	0.001	2.512 (1.009, 6.254)	0.048	1.676 (0.845, 3.327)	0.140	1.901 (0.978, 3.368)	0.058

The unadjusted and multi-adjusted hazard ratios of death were assessed for SF-12 and SF-36 quartile scores (Table 3). After multiple adjustment, for the PCS-12, patients with the lowest quartile score had a 2.5-fold higher risk of death compared to patients in the highest quartile i.e., the best perceived state. For the PCS-36 quartiles, the corresponding difference in risk was 2.7 after multiple adjustments.

The unadjusted and multi-adjusted HRs of death were also assessed for continuous SF-12 or SF-36 component summary scores (Table 4). During the follow-up, a one-unit increase in the PCS-12 score was related to 3.2% lower adjusted HR of death; a one-unit increase in the PCS-36 score was related to 2.3% lower adjusted HR of death.

**Table 4:** Unadjusted and multi-adjusted hazard ratios (HRs) for mortality were assessed for patients on dialysis (n = 252) grouped by continuous physical and mental component scores (PCS-36, MCS-36, PCS-12, and MCS-12), based on the SF-36 and SF-12

	**Unadjusted HR (95% CI)**	**p-value**	**Adjusted HR (95% CI)**	**p-value**
PCS-36 (per one increment unit)	0.963 (0.943, 0.984)	0.001	0.977 (0.953, 1.002)	0.077
PCS-12 (per one increment unit)	0.954 (0.931, 0.977)	<0.001	0.968 (0.942, 0.995)	0.022
MCS-36 (per one increment unit)	0.989 (0.970, 1.008)	0.248	0.995 (0.976, 1.015)	0.649
MCS-12 (per one increment unit)	0.981 (0.961, 1.001)	0.057	0.989 (0.968, 1.011)	0.339

The PCS-36 and PCS-12 were adjusted for age, gender, Charlsons comorbidity index without age, log transformed dialysis vintage, and albumin. The MCS-36 and MCS-12 are adjusted for age, gender, Charlsons comorbidity index without age, log transformed dialysis vintage, and hemoglobin.

## Discussion

We found that poor self-assessed physical health was an independent predictor of mortality in Norwegian patients on dialysis, after adjusting for established risk factors. This was consistent with results previously shown in other populations [11-14]. Beyond the confirmatory observation that low self-perceived physical aspect of HRQOL score is associated with higher risk of death, our results expand that finding that SF-12, as well as SF-36 revealed the increased mortality risk. In our study, one unit increase in PCS-12 score predicted 3.2% decreased adjusted HR of death, and one unit of increase in PCS-36 score 2.3% decreased adjusted HR of death. The great advantage of using SF-12 is that it comprises fewer items, it is less time-consuming, and easier to use, and thus, may represent a more clinically applicable tool for monitoring HRQOL. The latter observation was in accordance with the recent US study reporting that each incremental PCS-12 and PCS-36 point was associated with a 2.4% lower adjusted HR of death during a one year follow-up [21]. In our study, the adjusted HR of death was tripled, in patients in the lowest PCS-12 quartile compared to those in the highest quartile over the three to four-year period. The findings support the concept that a poor self-assessed HRQOL is an important risk factor for death, and it should not be ignored. Thus, measurement of HRQOL should be included in the general clinical work-up and follow-ups of patients on dialysis.

In contrast to some [12,13,15], but not all [11,16] other studies, we did not find any significant association between self-assessed mental health and mortality. Although we observed 1.1% reduction in the hazard ratio of death for every one-unit increase in MCS-12, this was not statistically significant. However, the magnitude was consistent with the 1.2% reduction in the adjusted hazard ratio of death recently reported by a large US study on patients on chronic dialysis [21]. The sample size in our study was most likely too small to reveal a significant relationship between death and MCS. Conflicting results have been reported in the literature on the effect of mental health on mortality. Nevertheless, the mental health effect has consistently been less than the effect of self-perceived physical health. Although the level of self-perceived mental health in the general population may differ among countries, the MCS scores in the large US study population [21] were similar to the MCS in our study population, and they observed that MCS as well as PCS predicted mortality. In this study, we excluded patients with cognitive disturbance, psychosis or drug-abuse. This exclusion may have affected the level of self-perceived mental health in our population, and could have led to a lower likelihood of predicting mortality. In at least some studies, a poor MCS score has been related to higher levels of depression, and depression has been shown to predict mortality in patients on chronic dialysis [27,28].

As suggested by Ware et al. [29], the use of SF-12, either interspersed within the SF-36, or on its own, has shown excellent correlations to the SF-36. The strong correlations that we observed between the SF-12 and SF-36 summary scores were consistent with findings in the general Norwegian population [30]. A recent cross-validation of the selected items for SF-12 was conducted in nine European countries; this led to the conclusion that data from the SF-12 were comparable to standard benchmarks [30]. Thus, our data extend that finding to include patients on chronic dialysis.

Some clinical and demographic characteristics of our study population were notable. The prevalence of diabetes in our study population was 26%, which is lower than that reported in other HRQOL studies; e.g., 66% was reported in the Spanish CALVIDA study [15], and nearly 50% was reported in a recent US study [21]. Diabetes has been a less prevalent cause of renal disease in Norwegian patients with ESRD compared to US patients on chronic dialysis [31]. Furthermore, in our study, the patients had undergone regular dialysis over a shorter period than that reported in other studies [15,21]. This was due to the high renal transplantation rate in Norway [32,33].

### Strengths and limitations of the study

One of the strengths of this study was that the sample was fairly large; it comprised close to one-third of the total population on regular dialysis in Norway at the time of sample selection [34]. In addition, the participation rate in the health survey was high, and none was lost to follow-up. The multi-center design ensured inclusion of patients from both rural and urban areas. Furthermore, socioeconomic status did not affect the possibility of dialysis. The characteristics of our patient population were quite similar to those of the general Norwegian population of patients on dialysis [34] in age, gender, and cause of renal failure. However, a selection bias could not be excluded, because the healthiest patients, both physically and mentally, might be more likely to participate in the study. Our data may underestimate the effect of HRQOL on clinical outcome, as patients with psychosis, drug abuse, cognitive disturbances, or recent hospitalization due to serious medical conditions were excluded. In this study we were committed to use the SF-36 version 1, in order to compare our results with Norwegian reference population [22,24]. Complete component summary scores could not be calculated for 10 patients in the SF-36 and for an additional 18 in the SF-12, due to missing single items. Only seven of the 301 patients were non-Caucasians; thus, the results may not be applicable to other populations. Furthermore, the renal transplantation rate in Norway is among the highest in Europe [32,35]. This affected the total time spent on chronic dialysis. During follow-up, 47% of patients received a kidney transplant in this study.

## Conclusions

Self-assessed physical health based on either the PCS-12 or PCS-36 is a strong, independent predictor of mortality in patients on chronic dialysis. The PCS-12 and PCS-36 provided comparable results. Thus, the physical aspects of HRQOL may increase the accuracy of risk stratification by adding important prognostic information for patients on dialysis. We suggest that the HRQOL assessment should be included in clinical investigations. Because the SF-12 requires less time to complete than the SF-36, it should be used routinely to assess HRQOL, in addition to the traditional, risk factors. It remains to be determined whether specific interventions aimed to improve HRQOL would affect the composite scores of either SF-12 or SF-36 and translate to improved survival.

## Competing interests

The authors declare that they have no competing interests.

## Authors’ contributions

IO, TD, IHN and LS prepared the study protocol and designed the study. TBHØ and VTP collected the data. TL provided data from the Norwegian Renal Registry on mortality and transplantation. TBHØ drafted the manuscript. TBHØ conducted the statistical analyses supervised by LS. TBHØ and IO interpreted the results. All co-authors critically reviewed the manuscript for important intellectual content and approved the final version to be published.
